# An increase in widespread extreme precipitation events during the northeast monsoon season over south peninsular India

**DOI:** 10.1038/s41598-023-50324-9

**Published:** 2023-12-20

**Authors:** Namendra Kumar Shahi, Shailendra Rai

**Affiliations:** 1 Institut des Géosciences de l’Environnement (IGE), Université Grenoble Alpes (UGA), 38400 Grenoble, France; 2 K. Banerjee Centre of Atmospheric and Ocean Studies, University of Allahabad, Prayagraj, India

**Keywords:** Climate sciences, Hydrology, Natural hazards

## Abstract

While the spatio-temporal characteristics of Indian summer monsoon precipitation and its extreme spells have been extensively studied, the northeast monsoon, which occurs from October to December (i.e., post-monsoon season) and affects the southern peninsula of India, has not received as much attention. In light of this, the present study explores the spatio-temporal characteristics of precipitation during the northeast monsoon, with a particular emphasis on widespread extreme precipitation events and their associated large-scale synoptic systems, using recent ensemble of high-resolution regional climate models (RCMs) simulations and the Indian monsoon data assimilation and analysis (IMDAA) reanalysis. The study reveals that both models tend to underestimate the intensity and frequency of observed precipitation events, although their skills in reproducing the observed spatial patterns of both mean and extreme precipitation are quite high (r > 0.75). A substantial increase in widespread extreme precipitation events (nearly twofold), along with a 30% rise in precipitation intensity, has been observed in the recent decade compared to the 1980s, and models demonstrate a similar directional change but tend to underestimate the magnitude of observed precipitation. This increase appears to be linked to the rapid warming of the Indian Ocean, which, in turn, increases the water vapor in the atmosphere, ultimately supplying more moisture to the southeastern peninsular India. On the other hand, observed discrepancies in replicating some of the reported widespread impactful extreme precipitation events in the years 2007 and 2015 over the southern India region underscore the need for caution when interpreting model simulations. Low-pressure systems, such as troughs, associated with cyclonic circulations originating from the Bay of Bengal, have been identified as the primary sources of moisture fueling heavy precipitation during these events. Cluster analysis highlights varying synoptic patterns within the general framework, emphasizing the need for a more nuanced approach in simulating and forecasting extreme precipitation events. Overall, this study underscores the importance of enhancing modeling capabilities to better understand and prepare for the growing challenges posed by extreme precipitation events.

## Introduction

The spatio-temporal characteristics of the monsoon precipitation makes the densely populated South Asian subcontinent one of the most vulnerable regions in the world to the impacts of natural disasters such as droughts and floods. The southwest monsoon or summer monsoon, which occurs from June to September, and the northeast monsoon or winter/post monsoon, which takes place from October to December, are the two main monsoonal systems of South Asia^1–4^. While the southwest monsoon accounts for a major portion of the annual precipitation over India, the precipitation during the northeast monsoon season is also crucial, especially for the southern peninsula India. In fact, the southern peninsula of India remains under the rain-shadow region during the southwest monsoon season^2,4^. While various aspects of the southwest monsoon have been extensively studied, the northeast monsoon has received comparatively less attention. Highly impactful extreme precipitation events and the resulting severe socio-economic consequences are common over parts of India during both monsoonal systems. In fact, extreme precipitation events, resulting in flash floods and landslides, are among the most devastating natural hazards in India. These extreme events pose significant risks and have far-reaching impacts to life, property, and the economy. Inland flooding events in India result in economic losses of approximately $3 billion per year, accounting for 10% of global economic losses^5^. Therefore, a better understanding and more accurate prediction of extreme precipitation events is vital to reduce the risks associated with these extreme events.

Extreme precipitation events are more frequent in the plains of central India, i.e., the flood-prone region, during the summer monsoon season^5,6^, while the region of southeastern peninsular India experiences extreme events during the northeast monsoon season^7,8^. Previous studies have reported a significant increase in the frequency and intensity of extreme precipitation events over central India during the summer monsoon season^5,6^, and is attributed to the increasing variability of the low-level monsoon westerlies over the Arabian Sea, which drives surges of moisture supply, resulting in extreme precipitation episodes across the central India^5^. During the northeast monsoon season, depressions and cyclonic storms occasionally form over the southern Bay of Bengal ocean and are associated with extreme precipitation over southeastern peninsular India^7^. However, the spatio-temporal characteristics of extreme precipitation events and their associated synoptic-scale conditions during the northeast monsoon have not been comprehensively explored on climate-scale, or we can say, has received less attention. With this motivation, our main goal here is to explore the spatio-temporal variability of precipitation, with the main focus on extreme precipitation events.

In terms of the modeling aspect, it has been observed that climate models poorly simulate the mean state and spatio-temporal variability of northeast monsoon precipitation^2,4,9,10^. Therefore, it is important to investigate the ability of recent high-resolution simulations and reanalysis datasets in representing the spatio-temporal variability of northeast monsoon precipitation. In light of this, the recent high-resolution Indian Monsoon Data Assimilation and Analysis reanalysis (IMDAA) and recent CORDEX-CORE regional climate model simulations^11,12^ have been analyzed in this study.

## Results

### Mean precipitation

**Figure 1:**
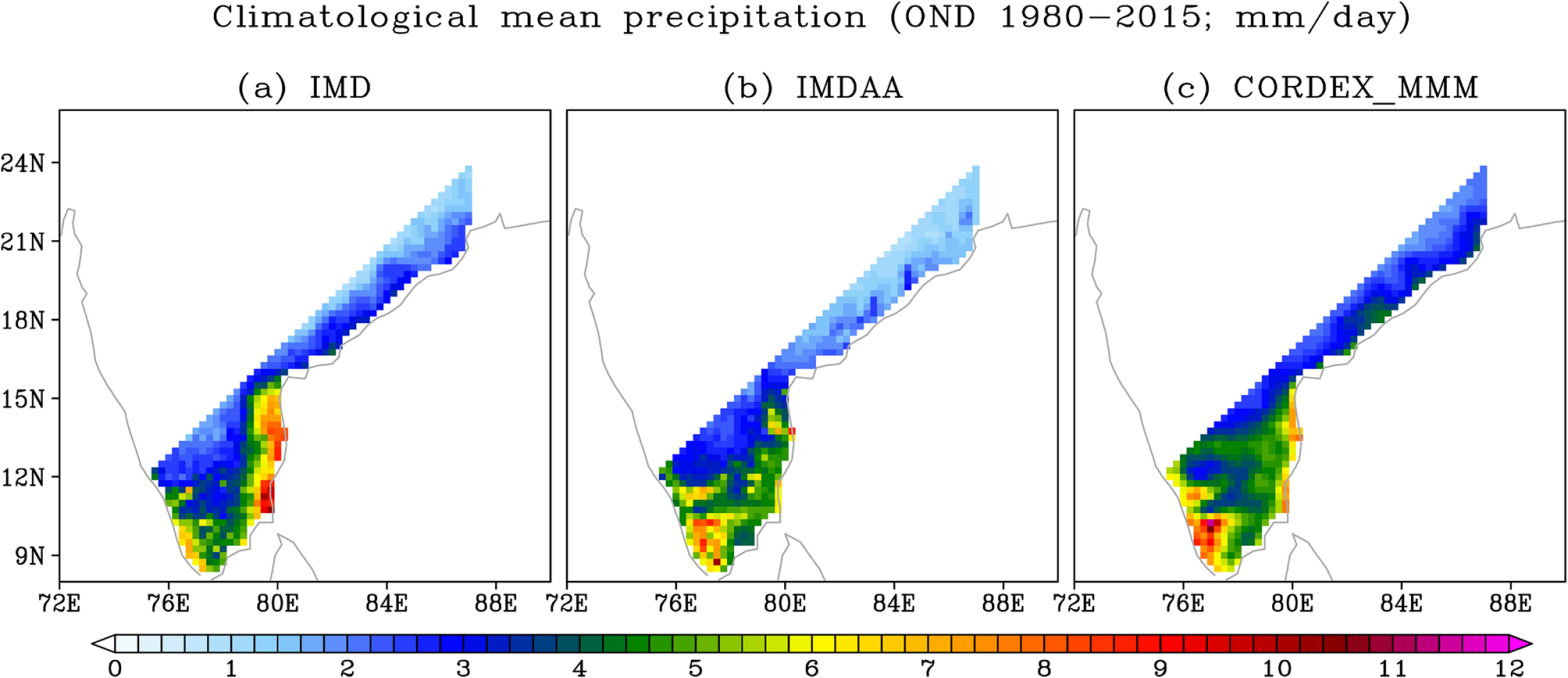
Climatological (1980–2015) OND seasonal mean precipitation from (**a**) IMD, (**b**) IMDAA, and (**c**) CORDEX_MMM. Units are in mm day^-1^.

**Figure 2:**
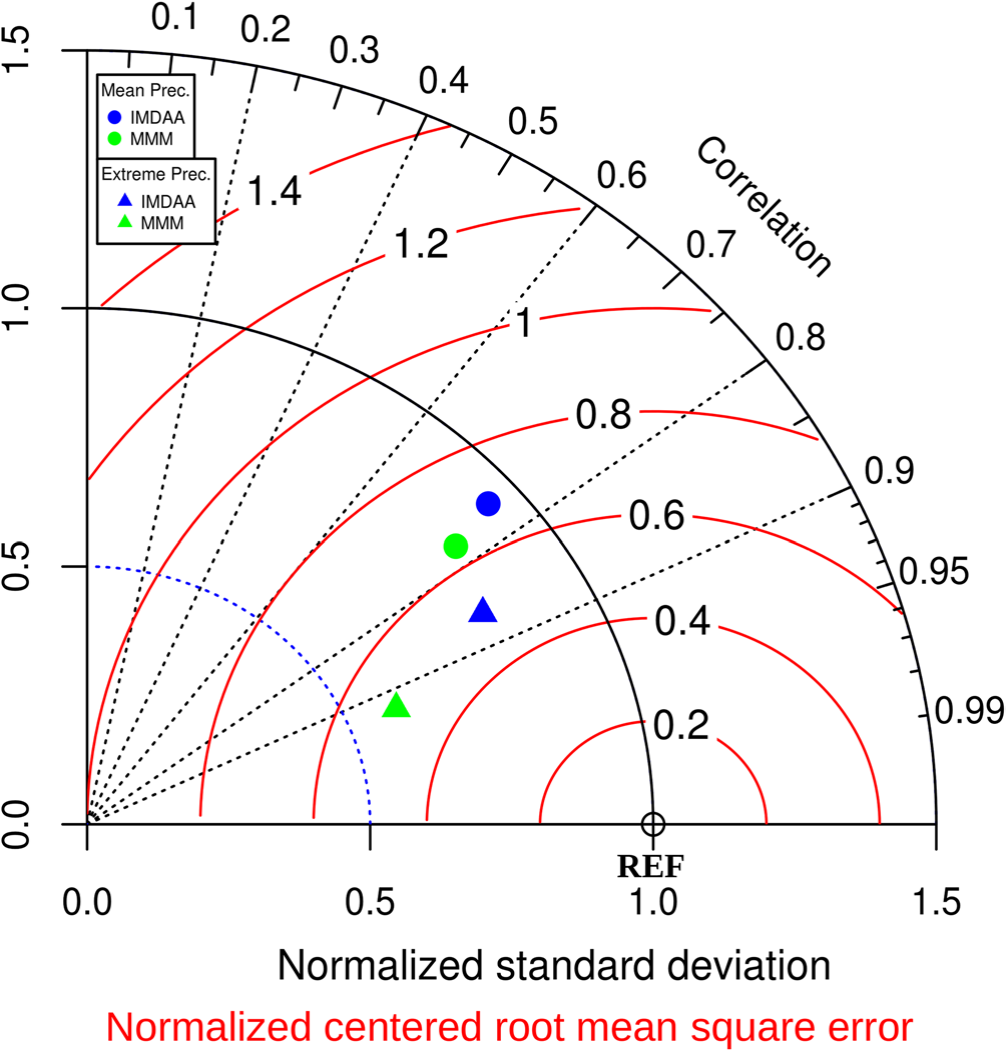
Taylor diagram for the seasonal mean precipitation and extreme precipitation (i.e., precipitation intensity during the widespread extreme events). The diagram combines the spatial correlation (black, dashed line), root mean square error (RMSE; red, solid arcs), and standard deviation (SD; blue, dashed arcs). The RMSE and SD of the individual models are normalized by the observed spatial SD.

We commence the analysis by assessing the seasonal climatological mean precipitation over the NEM region using both observations and models for the period 1980–2015, as illustrated in Fig. 1. The Taylor diagram has been utilized to assess the performance of simulations^13^, as shown in Fig. 2. The maximum precipitation is located along the coastal region, extending/stretching from southern Kerala in the west to southern Andhra Pradesh in the east, with a gradual decrease inland. The noticeable overestimation (underestimation) of precipitation by models along the western coast including inland areas (eastern coast) can be observed. Additionally, overestimation and underestimation of precipitation by RCMs and IMDAA, respectively, are observed over the north-eastern region. Despite these biases, both models demonstrate a high skill in capturing the observed spatial patterns, with correlation values exceeding 0.75 (Fig. 2).

**Figure 3:**
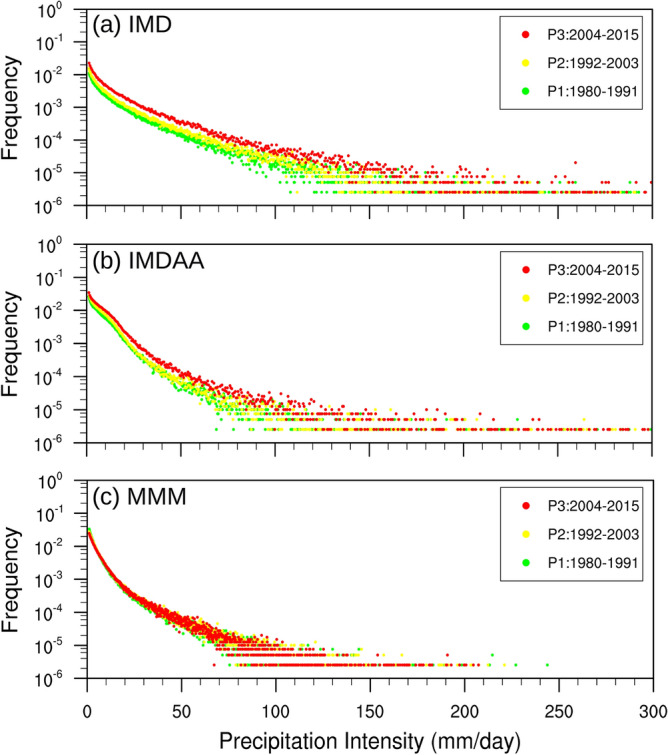
Probability density function (PDF) of daily mean precipitation in the study region located below 17° N over three 12-year time spans from (**a**) IMD, (**b**) IMDAA, and (**c**) CORDEX_MMM. Units are in mm day^-1^.

We have extended our analysis by calculating and plotting the PDF over three 12-year time spans, rather than over the entire period (i.e., 36-year), in order to assess whether there have been any changes in the frequency and intensity of precipitation in this region over time (Fig. 3). The PDF is a widely-used technique for visualizing the distribution of precipitation. Here, we have computed the PDF using the each grid cells information of study domain for precipitation intensity exceeding one mm. Our analysis reveals a clear increase in both the frequency and intensity of precipitation in recent decades compared to previous decades, as evidenced by the observed tail. This increase may be attributed to climate change. The observed increasing signal is also simulated by IMDAA reanalysis, although this signal is not well-replicated by RCMs. This discrepancy raises questions about the confidence we can place in the future scenarios simulated by RCMs, given their inability to faithfully reproduce the observed signal during the evaluation run.

**Figure 4:**
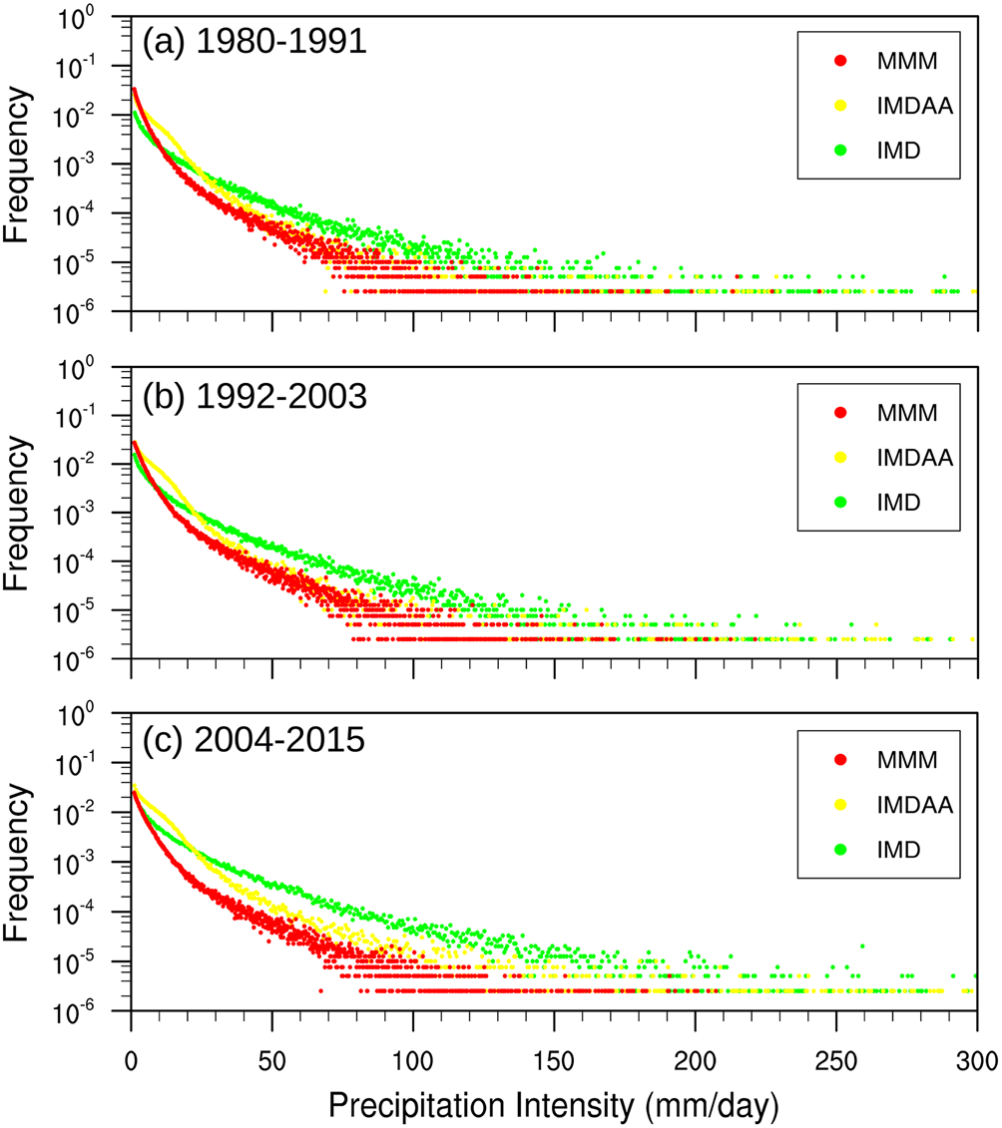
Probability density function (PDF) of daily mean precipitation in the study region located below 17° N from IMD, IMDAA, and CORDEX_MMM over three 12-year time spans. Units are in mm day^-1^.

The comparison between the observed tail and the tails simulated by the models has been conducted to assess how accurately the models represent the observed tail (Fig. 4). It has been observed that IMDAA tends to overestimate low-to-moderate-intensity precipitation events (< = 25) and underestimate precipitation events exceeding 25 mm for all three time spans. In the case of RCMs, overestimation has been observed for precipitation events less than 10 mm, while underestimation is noted for precipitation events greater than 10 mm. The excess in occurrence of precipitation events with low (< = 5 mm) and moderate (> = 30 and <  = 80) intensity can be clearly observed. Additionally, it is worth noting that overestimation decreases and underestimation increases when transitioning from the last decades to the current decades. On the other hand, the observed overestimation and underestimation of frequency as well as intensity of precipitation in the models can explain the distribution of mean precipitation distribution^14,15^.

### Characteristics of Widespread extreme precipitation events

In this section, we have examined the characteristics of widespread extreme precipitation events and their associated synoptic-scale features. The identification of widespread extreme precipitation events has been carried out for each datasets using the method outlined in the methods section. The event identification has been conducted in the study region located below 17° N, as the highest mean and extreme precipitation have been observed in this region (e.g., Figs. 1 and 6). Also, the IMDAA reanalysis has been regridded onto the CORDEX grid before applying the criteria to identify widespread extreme precipitation events. On the other hand, due to the unavailability of datasets for many variables in the majority of RCM simulations in the CORDEX public domain (e.g., the unavailability of winds and humidity data at pressure levels limits our analysis of vertically integrated moisture transport and its convergence), we have endeavored to examine the synoptic-scale features during widespread extreme precipitation events using the 850-hPa wind and 500-hPa geopotential height. Although, we have calculated the vertically integrated water vapor transport and its convergence for IMDAA reanalysis to assess the source of moisture supply in the study domain during the widespread extreme precipitation events.

### Event statistics

**Figure 5:**
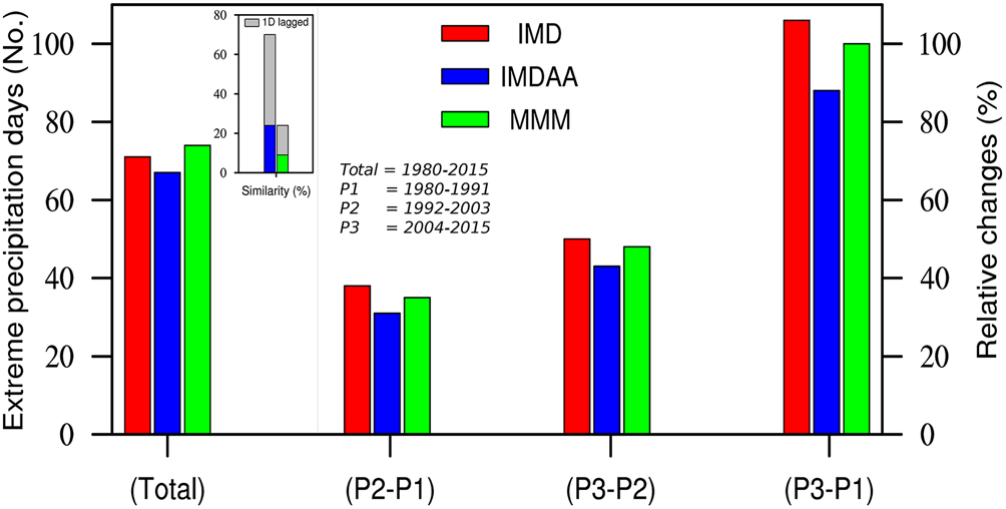
Total number of widespread extreme precipitation events (in No.), similarity with observed widespread extreme precipitation events (in %), and changes in widespread extreme precipitation events (in %).

**Figure 6:**
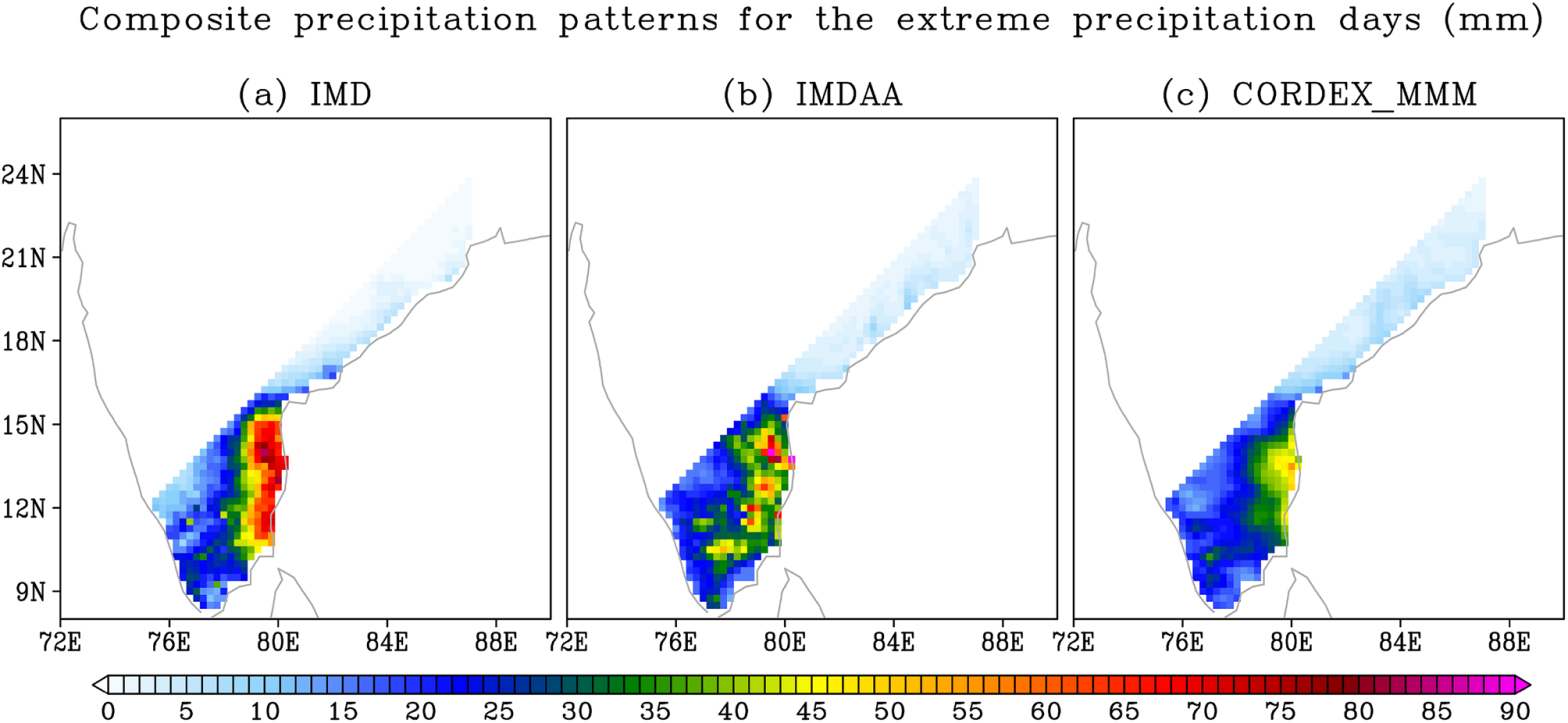
Composite of mean precipitation for widespread extreme precipitation events/days. Units are in mm day^-1^.

We commence our analysis of extreme precipitation events by examining the events themselves and their fundamental statistics. The total number of widespread extreme precipitation events is found to be 71 for observations, 67 for IMDAA, and 74 for MMM (Fig. 5). Furthermore, a composite of daily mean precipitation for each dataset has been made using their respective widespread extreme events/days, as shown in Fig. 6. The highest precipitation during extreme events is observed along the eastern coastal region in both observations and models (Fig. 6). However, models tend to underestimate the intensity of precipitation, with the maximum underestimation observed in the case of MMM (Fig. 6). The skills of models in reproducing the observed spatial pattern of precipitation are quite high, with correlation values exceeding 0.85, although the underestimation of spatial variability is obvious (Fig. 2).

Furthermore, we observed an increase of approximately 40% in extreme events between the 1992–2003 (P2) and 1980–1991 (P1) periods and approximately 50% in the recent decade compared to the previous one (Fig. 5). A substantial increase (approximately twofold or 100%) in extreme events in the recent decade compared to the 1980s is clearly evident (Fig. 5). Despite MMM's inability to accurately reproduce the observed tails, as we noted (i.e., Figs. 3 and 4), it has reasonably well represented the characteristics of observed extreme precipitation events. A slight underestimation can be observed in the case of IMDAA in simulating extreme events and in decadal changes when compared to the observations (Fig. 5). On the other hand, not much increase (on average of 5%) has been observed in precipitation intensity during extreme events between the P2 and P1 time periods, while on average increase of 30% has been observed in the recent decade compared to the P1 time period (Fig. not shown). Overall, we observed a significant increase in extreme events with precipitation intensity in recent decades compared to the 1980s, and models show a similar directional change but tend to underestimate the magnitude of precipitation. On the other hand, it has been noted that IMDAA correctly simulated approximately 25% of the observed event dates, while MMM reproduced only about 10% of the observed events (Fig. 5). However, these percentage increases by 45% and 15% for IMDAA and MMM, respectively, when considering the model's performance, taking into account a one-day lagged (lead/lag) in the observed dates (Fig. 5).

**Figure 7:**
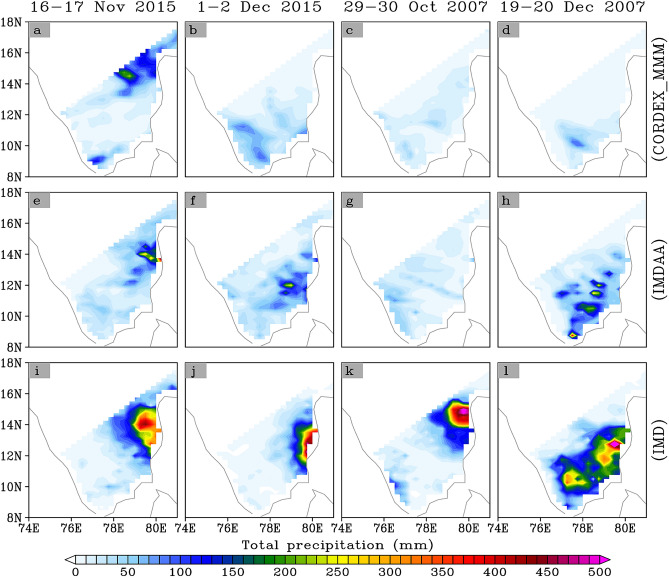
Total precipitation during some of the reported widespread and impactful historical flood events.

As we noted, only about the 25/10% (up to 70/25% with one-day lagged) of observed extreme days are correctly reproduced by the IMDAA/MMM. Therefore, it is crucial to analyze some reported widespread and highly impactful extreme precipitation events in this region to determine if the models are capable of simulating these events. The widespread heavy precipitation events during November 16–17, 2015, and December 1–2, 2015, occurred over the coastal region of southern Andhra Pradesh and Tamil Nadu, including Chennai, as reported by IMD, has been considered for this analysis. During November–December 2015, the coastal regions of the south Indian states of Tamil Nadu and Andhra Pradesh experienced catastrophic flooding events. These events resulted in widespread flooding in the area, displacing over 1.8 million people with More than 500 people were killed and affecting a total of 4 million individuals, with the estimated damages and losses amounted to 3 billion U.S.D.^16,17^. In addition to the aforementioned events, we have also analyzed two further occurrences reported by the IMD (i.e., October 29–30, 2007 and December 19–20, 2007). During 29–30 October, heavy rainfall was observed over southern Andhra Pradesh with the peak over Nellore and surrounding areas (21 causalities were reported), while during 29–30 October, heavy rainfall occurred over Tamil Nadu with the peak over Chennai (30 causalities were reported) [https://imdpune.gov.in/library/public/Disastrous%20Weather%20Events%202007.pdf]. We have plotted the spatial pattern of total precipitation during all four events as shown in Fig. 7. It can be seen from the figure that the model completely fails to replicate the observed precipitation distribution during all selected extreme events, raising concerns about our confidence in the recent IMDAA reanalysis.

### Synoptic composites

**Figure 8:**
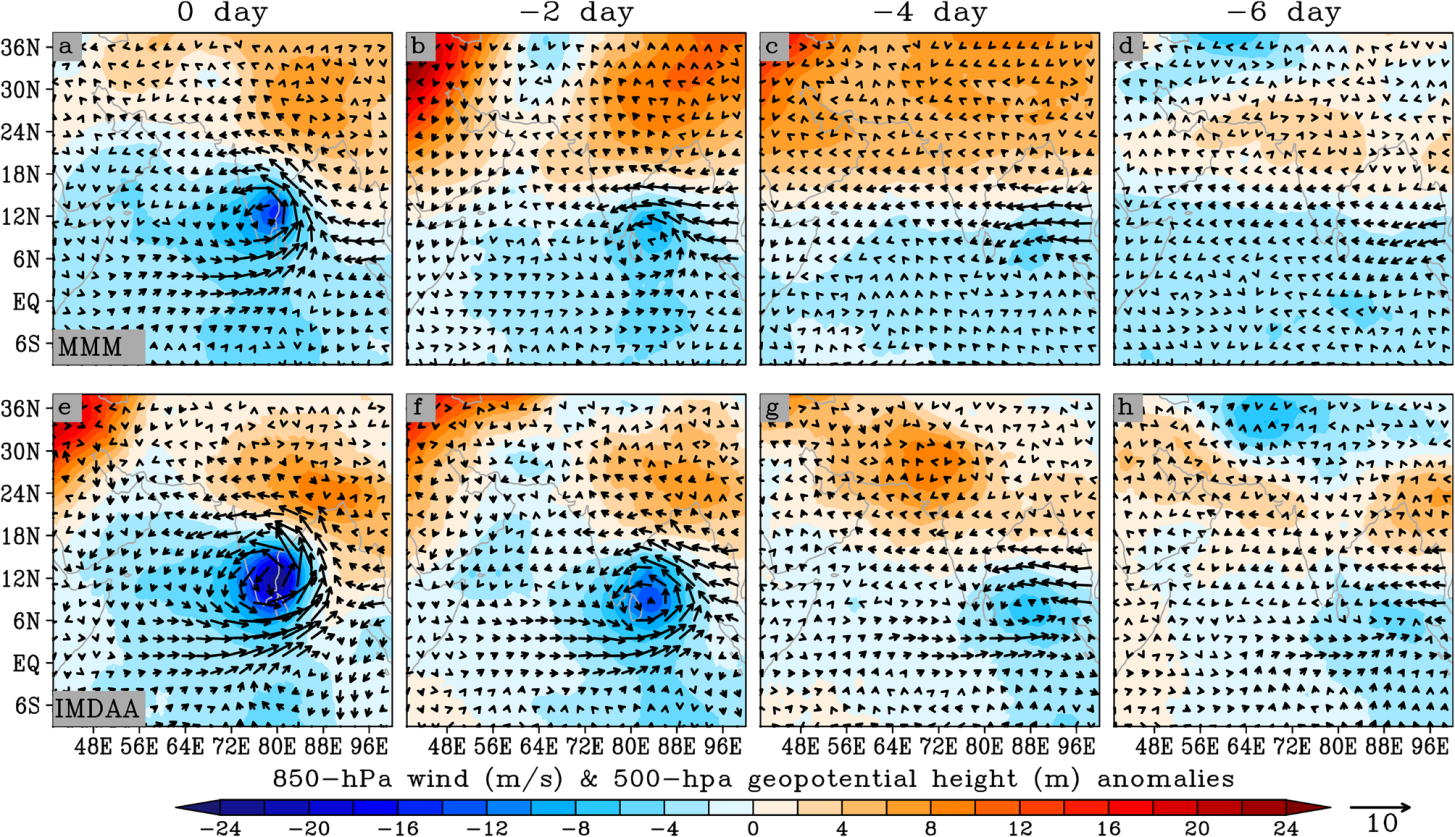
Composite of 500-hPa geopotential height (in meters; shaded), and 850-hPa wind vectors (in m/s; arrows) for widespread extreme days. The upper and lower panels show results obtained from CORDEX_MMM and IMDAA, respectively.

**Figure 9:**
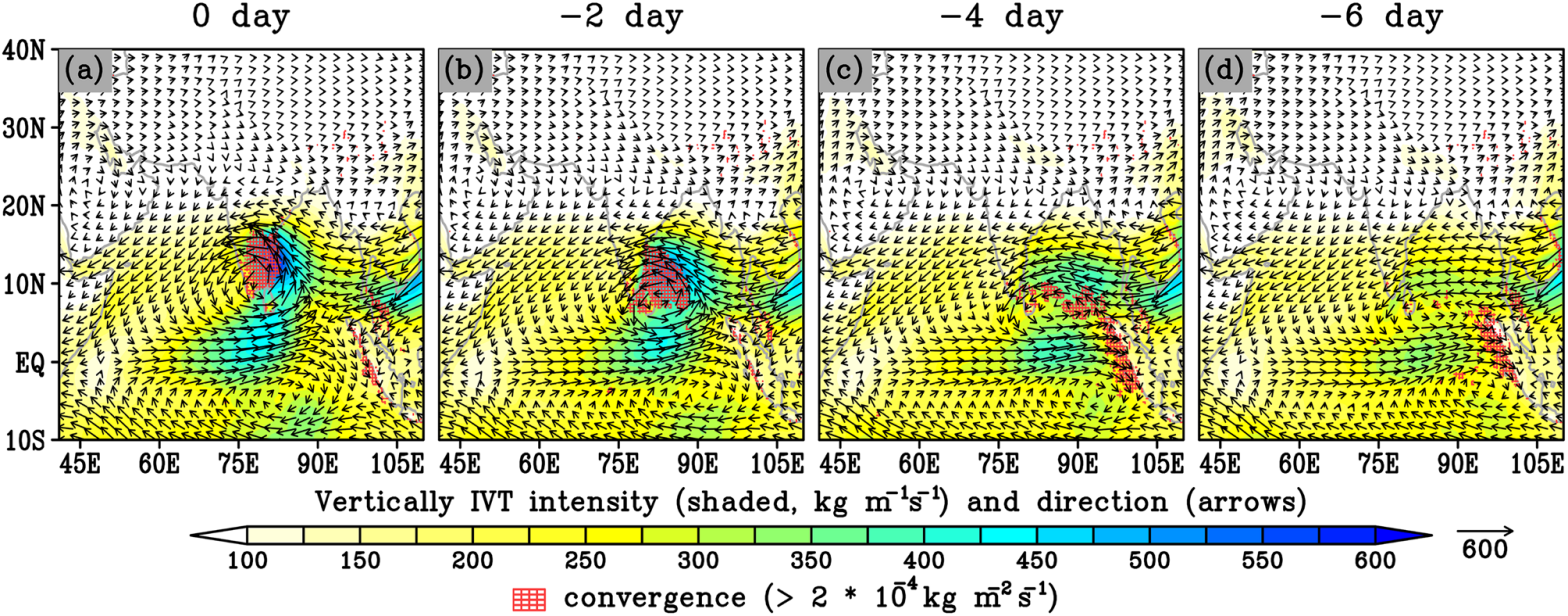
Composite of vertically-integrated mean water vapor transport (IVT) magnitude (shaded, kg m^-1^ s^-1^) and direction (arrows), and convergence (> 2 × 10^–4^ kg m^-2^ s^-1^) indicated by red hatches for widespread extreme days from IMDAA.

To further understand the state of the atmosphere during extreme precipitation events, and identify the physical processes involved, we composite daily anomalies of 850-hpa wind and 500-hpa geopotential height over the entire period of obtained extreme days, as shown in Fig. 8. To comprehend the evolution of synoptic conditions that lead to extreme precipitation events and to identify synoptic patterns and precursors ahead of such events, composite maps have been prepared for up to six days prior to the peak event, which could lead to an improvement in the skill of forecasting these events (Fig. 8). Furthermore, the vertically integrated moisture transport and its convergence for the IMDAA datasets has also been computed as shown in Fig. 9. The development of a upper level trough-ridge dipole-like pattern, with the trough over the southern Bay of Bengal oceanic region and a ridge near northeast India, can be clearly observed from the IMDAA six days prior to the extreme events (Fig. 8). This trough, characterized as a low-pressure system, is associated with the formation of a low-level cyclonic circulation with southwesterly winds from the southern Indian Ocean and easterly winds from the western Pacific Ocean region (Fig. 8). The trough associated with the low-level cyclonic pattern strengthens and moves northwestward as we approach the day when extreme precipitation occurred (Fig. 8). On the day of the extreme precipitation event, a pronounced trough with a strong cyclonic circulation centered over the southern India region and a ridge pattern over the northeast India have been well established (Fig. 8). The established cyclonic pattern over southern India resulted in heavy rainfall in this region. The transport of moisture from the western Pacific Ocean (carried by the easterly flows) with the contributions from the central Indian ocean through westerly flows over the southern India, which contribute to the convergence over the region (resulting into precipitation), can be clearly observed from the Fig. 9. Overall, we can conclude that the low-pressure systems (i.e., trough) which develop in the Bay of Bengal and move northwestward into southern India are a major source of moisture for heavy precipitation. As the IMDAA demonstrates a closer agreement in intensity and distribution of precipitation with observations during extreme events, the synoptic pattern observed in the IMDAA can be considered the true condition. In the case of MMM, a slight difference can be observed in spatial trough-ridge patterns with a weaker evaluation of the low-level cyclonic circulation compared to the IMDAA (Fig. 8). This variation could potentially explain the slight differences observed in precipitation between the two datasets.

### Clustering of synoptic composites

On the mean scale, it is now evident that the development of cyclonic circulation in the Bay of Bengal and its northwestward movement into southern India is a major source of moisture for heavy precipitation in the southern India region. However, it could be possible that some of the extreme event cases may have a different synoptic pattern than what we observed in the case of the mean condition. Also, it is obvious that the pattern cannot be entirely different from the observed mean condition, but it could at least be somewhat distinct. To investigate this, a k-means cluster analysis with a cluster size of 4 has been conducted on the anomalous 500-hPa geopotential height field. The cluster analysis is performed on all 67 and 74 extreme days for IMDAA and MMM, respectively. The resulting four cluster composites, along with their frequencies of occurrence (%), are shown in Fig. S1 for IMDAA and Fig. S2 for MMM. The low-level winds are also plotted with the 'day' associated with a particular cluster occurrence. In all four cluster analyses, the presence of low-level cyclonic circulation associated with an upper-level trough over the southern India region and its origin from the Bay of Bengal are evident (Fig. S1). However, differences can be observed in the spatial pattern of synoptic patterns compared to the mean pattern. For example, an opposite pattern can be observed with high height anomalies in clusters 2 and 4, as well as in clusters 1 and 3 (Figs. S1 and 8). In the case of the MMM, a clear low-level cyclonic circulation over southern India can be observed in clusters 2–3, and it is closer to the IMDAA in terms of intensity and spatial distribution (Fig. S2). These two clusters (2–3) are associated with 60% of extreme days, while the remaining (40%) extreme days are associated with clusters 1–4 in the case of MMM (Fig. S2). Conversely, in the case of IMDAA, more extreme events are associated with cluster 1–4 (66%) compared to cluster 2–3 (34%) (Fig. S1). On the other hand, a pronounced cyclonic circulation with trough activity has been observed in the case of IMDAA during cluster 1–4; however, the observed pattern is not well-replicated by the MMM, and significant differences in spatial patterns can also be observed. The observed differences between IMDAA and MMM underscore the need for continued refinement and improvement in modeling techniques to enhance the accuracy and reliability of weather predictions in this region.

### Role of SST warming in the rise of extreme events

**Figure 10:**
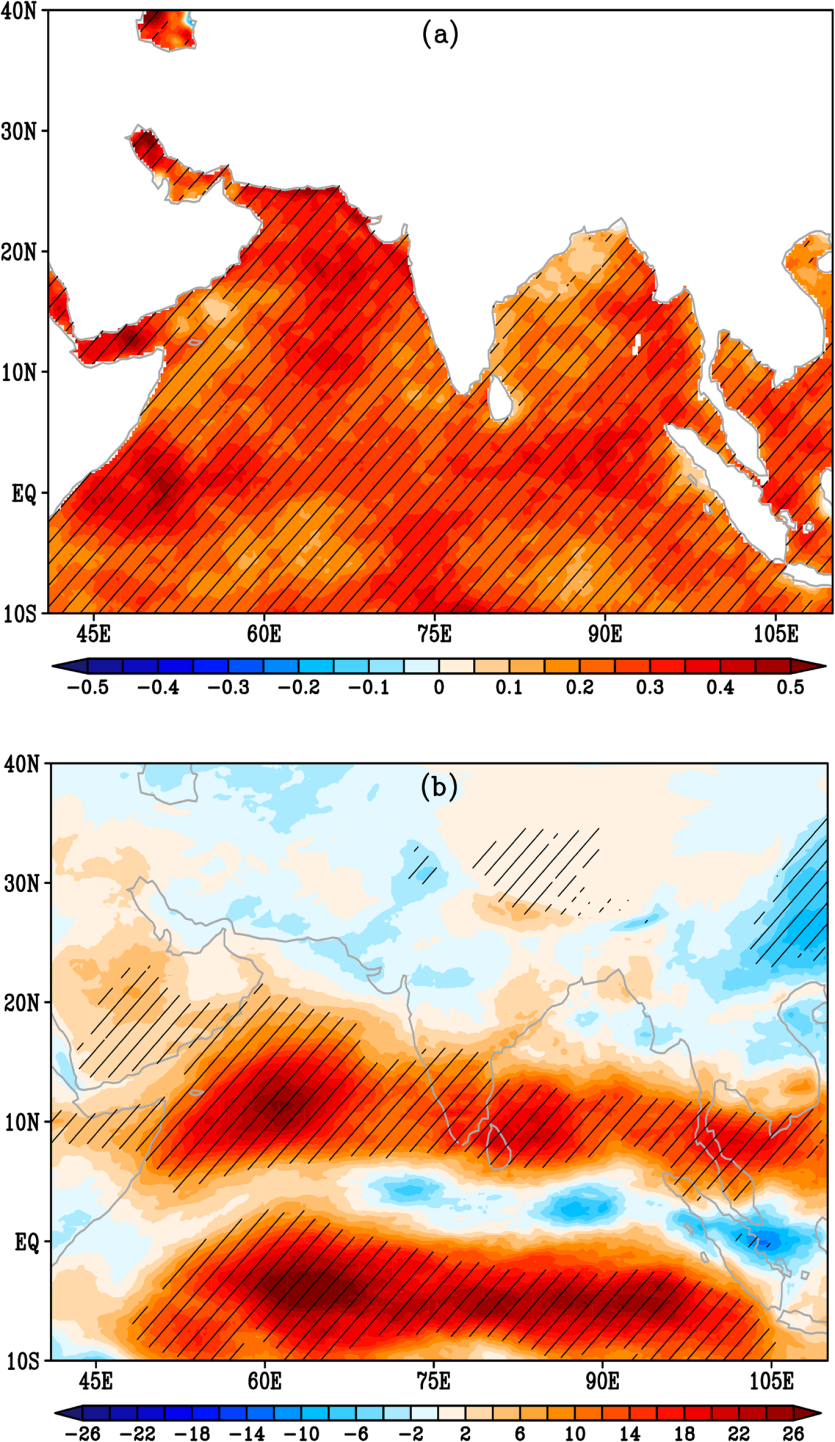
Spatial distribution of Theil-Sen trends for sea surface temperature (SST) anomalies and vertically-integrated mean water vapor transport (IVT) magnitude during OND 1980–2015: [**a**] Trends for seasonal SST anomalies (°C per decade). [**b**] Trends for seasonal mean IVT magnitude (kg m^-1^ s^-1^ per decade). Black hatches represent areas with statistically significant trends above the 95% confidence level (Mann–Kendall sign above 0.95). This analysis utilizes the NOAA high-resolution (0.25° × 0.25°) version 2 daily SST dataset.

In this section, we aim to investigate the potential causes of the observed twofold increase in widespread extreme precipitation events as well as a significant rise in the frequency and intensity of daily precipitation amounts in the recent decade compared to the 1980s. We speculate that this increase could possibly be due to an increase in SST caused by global warming. It has been well established that increases/warms in SST have led to an increase in atmospheric water vapor, ultimately influencing entire weather systems and increasing the risk of more frequent heavy precipitation events^18^. To explore this, we have analyzed the trends in seasonal SST anomalies and the magnitude of vertically integrated mean water vapor transport (IVT) during the period from 1980 to 2015, as shown in Fig. 10. The Mann–Kendal—Theil-Sen trend analysis is used to assess the strength and significance of trends^19–21^. The significant increase in SST, up to 0.5 °C per decade, over the entire tropical South Asian region during 1980–2015 can be clearly observed. Furthermore, an increase in moisture content (up to 30 kg m^−1^ s^−1^ per decade) has been observed in bands extending from the western Pacific ocean to the Arabian sea region in both the northern and southern equatorial tropical regions. This rise in moisture from the western Pacific Ocean to the Arabian Sea region, possibly through the easterly flow from the western North Pacific Ocean, potentially leads to more frequent extreme precipitation events with an increase in precipitation intensity over southeastern Peninsular India in recent decades.

## Discussion

The present study explores the spatio-temporal distribution of precipitation characteristics, with a particular emphasis on widespread extreme precipitation events and their associated large-scale synoptic systems over the South India region during the northeast monsoon season (OND) using observational (i.e., IMD) and recent high-resolution regional climate model simulation datasets (i.e., IMDAA and RCMs). The study is centered on the time frame of 1980–2015. While models exhibited notable precipitation biases, such as overestimation along the western coast and underestimation along the eastern coast, they nevertheless demonstrated a strong ability to capture the overall observed spatial pattern of seasonal mean precipitation (correlation values exceeding 0.75). Comparing the observed tail of precipitation events with model-simulated tails highlighted the limitations of the models. IMDAA tended to overestimate low-to-moderate-intensity precipitation events and underestimate high-intensity precipitation events, while RCMs exhibited overestimation for low-intensity precipitation events and underestimation for moderate-to-high-intensity precipitation events. The observed overestimation and underestimation in both frequency and intensity of precipitation events in the models help explain the distribution of mean precipitation intensity. Further investigation into the temporal evolution of precipitation revealed a significant increase in both the frequency and intensity of precipitation in recent decades, suggesting a possible influence of climate change. This signal was partly reproduced by IMDAA datasets, but the RCMs struggled to replicate it accurately, raising concerns about their reliability for future climate scenarios. Overall, our findings emphasize the need for continued model refinement and caution when interpreting future projections, given the observed limitations in reproducing historical precipitation patterns in the NEM region.

Furthermore, the analysis of widespread extreme precipitation events and their synoptic characteristics, shedding light on the challenges and complexities associated with modeling and predicting such events. Models tend to underestimate the intensity of precipitation during widespread extreme precipitation events, although their skills in reproducing the observed spatial pattern of precipitation are quite high, with correlation values exceeding 0.85. A substantial increase in widespread precipitation extreme events (approximately double or 100%) with precipitation intensity (approximately 30%) in the recent decade compared to the 1980s has been observed, and models demonstrate a similar directional change but tend to underestimate the magnitude of precipitation. The rise in these events is possibly due to an increase in water vapor (due to SST warming) over the southeastern Indian Peninsula through the western Pacific and Indian Ocean regions. It has also been interesting to observe the decreasing trend in IVT in the zone between the southern and northern Indian Ocean. On the other hand, it has also been observed that the models completely fail to replicate some reported widespread impactful extreme precipitation events in the years 2007 and 2015 over the southern India region, raising concerns about our confidence in the model simulations. Further, we have provided valuable insights into the synoptic conditions leading to heavy precipitation events over the southern India region during the widespread extreme precipitation events. Our findings indicate that the low-pressure systems (i.e., trough) associated with a low-level cyclonic circulation develop in the Bay of Bengal and move northwestward into southern India serve as a primary source of moisture (i.e., enhanced moisture advection from Bay of Bengal as well as central Indian Ocean), fueling heavy precipitation during the widespread extreme precipitation events. On the other hand, the RCMs exhibits slight variations in spatial synoptic patterns and a weaker evaluation of the low-level cyclonic circulation compared to the IMDAA, which may account for the slight discrepancies in precipitation between the two datasets during the extreme events.

Furthermore, a k-means cluster analysis has been conducted to explore variations in synoptic patterns associated with widespread precipitation extreme events. While all clusters exhibited the presence of low-level cyclonic circulation and an upper-level trough originating from the Bay of Bengal, differences in spatial patterns are evident. The analysis has identified four distinct clusters, each with varying percentages of occurrence for IMDAA and MMM. These results highlight the potential for different synoptic patterns within the general framework observed in the mean condition, emphasizing the need for a more nuanced approach in forecasting extreme precipitation events.

In light of these findings, it is clear that there is a need for continued improvement in modeling capabilities, particularly in capturing the complexity and variability of extreme precipitation events. Understanding the limitations and strengths of these models is vital for making informed decisions related to disaster management, infrastructure planning, and climate change adaptation in the face of increasing extreme precipitation events. Future research should focus on refining model performance and addressing the challenges associated with simulating extreme precipitation events accurately.

## Methods

### Data

In this study, we employed the most recent high-resolution simulations (horizontal resolution of 0.22° × 0.22°) conducted over the South Asia domain as part of the World Climate Research Programme (WCRP)-sponsored Coordinated Regional Climate Downscaling Experiment-Coordinated Output for Regional Evaluations (CORDEX-CORE) project^22^. All simulations utilized in this study are evaluation runs based on ERA-interim reanalysis data spanning the period 1980–2015; further details can be found in Table 1. The observational data utilized in this study consists of gridded daily rainfall data from the India Meteorological Department (IMD) with a spatial resolution of 0.25° × 0.25°^23^. Additionally, we incorporated the recent high-resolution product i.e., Indian Monsoon Data Assimilation and Analysis reanalysis (IMDAA; 0.12° × 0.12°) from the National Centre for Medium Range Weather Forecasting (NCMRWF)^24^. The high-resolution daily optimum interpolation sea surface temperature (OISST; 0.25° × 0.25°) data from the National Oceanic and Atmospheric Administration (NOAA) has also been utilized^25^.

**Table 1:** Ensemble of RCM simulations used in this study. More detailed information about the model configurations can be found in ^22^.

Institution	Model	Reference
Climate Service Center (GERICS), Hamburg, Germany	REMO2015	^26^
Eidgenössische Technische Hochschule (ETH) universität, Zürich, Switzerland	COSMO-crCLIM-v1-1	^27^
The Abdus Salam International Centre for Theoretical Physics (ICTP), Trieste, Italy	RegCM4.7	^28^

### Identification of widespread extreme precipitation events and clustering analysis

The widespread extreme precipitation events/days are defined as days when precipitation exceeds the thresholds of R99p and R99p_reg_ in at least 20 grid cells of the study domain under consideration. Here, R99p represents the 99^th^ percentile of daily precipitation during SON 1980–2015, and R99p_reg_ is the area-averaged R99p value over the study domain. While the use of the R99p threshold is a common practice for defining extreme precipitation events, relying solely on it may not be very reliable when considering a significant number of grid cells/points to identify widespread extreme precipitation events. This unreliability arises from the possibility of very low R99p thresholds at some grid points. To address this concern, we also incorporate the threshold of R99p_reg_ along with the R99p threshold. Furthermore, we perform k-means clustering analysis using Euclidean distance on anomalous 500-hPa geopotential heights to identify large-scale circulation patterns associated with the widespread extreme precipitation events^29^.

### Moisture transport and flux convergence

Furthermore, vertically integrated water vapor transport (IVT) and vertically integrated moisture flux convergence (VIMFC) have also been calculated to investigate the total moisture influx into the study region during the widespread extreme precipitation events. These analyses are crucial components of studying extreme weather events, particularly those associated with heavy precipitation. The IVT and VIMFC are computed using the moisture budget equation over the troposphere (1000–200 hPa), which can be defined as:

$$IVT=\frac{1}{g}{\int }_{{P}_{200}}^{{P}_{1000}}qVdP$$

$$VIMFC=-\nabla .\frac{1}{g}{\int }_{{P}_{200}}^{{P}_{1000}}qVdP$$

where $\nabla \cdot \left(\right)$ is the horizontal divergence in pressure coordinates,$q$ is the specific humidity,$V=\left(u,v\right)$ is the wind vector,$P$ is the pressure, and $g$ is the acceleration due to gravity.

### Supplementary Information

Supplementary Figures.

## Data Availability

The RCM datasets utilized in this study are freely accessible on the World Climate Research Programme (WCRP) Coordinated Regional Climate Downscaling Experiment (CORDEX) website under ESGF (https://cordex.org/data-access/esgf/). The Indian Monsoon Data Assimilation and Analysis (IMDAA) reanalysis can be obtained from https://www.ncmrwf.gov.in/data/, and the IMD data is available at https://www.imdpune.gov.in. The daily optimum interpolation sea surface temperature (OISST) is freely accessible from the National Oceanic and Atmospheric Administration (NOAA) at https://psl.noaa.gov/data/gridded/.

## Code availability

The analysis codes supporting the findings of this study are accessible from the corresponding author upon a reasonable request.
