# Supplementary material for: An increase in widespread extreme precipitation events during the northeast monsoon season over south peninsular India
Source: Sci Rep. 2023 Dec 20;13:22757. doi: 10.1038/s41598-023-50324-9 (PMC10733342; doi:10.1038/s41598-023-50324-9)
Supplement: Supplementary file 1 — Supplementary Figures. [file 41598_2023_50324_MOESM1_ESM.pdf]

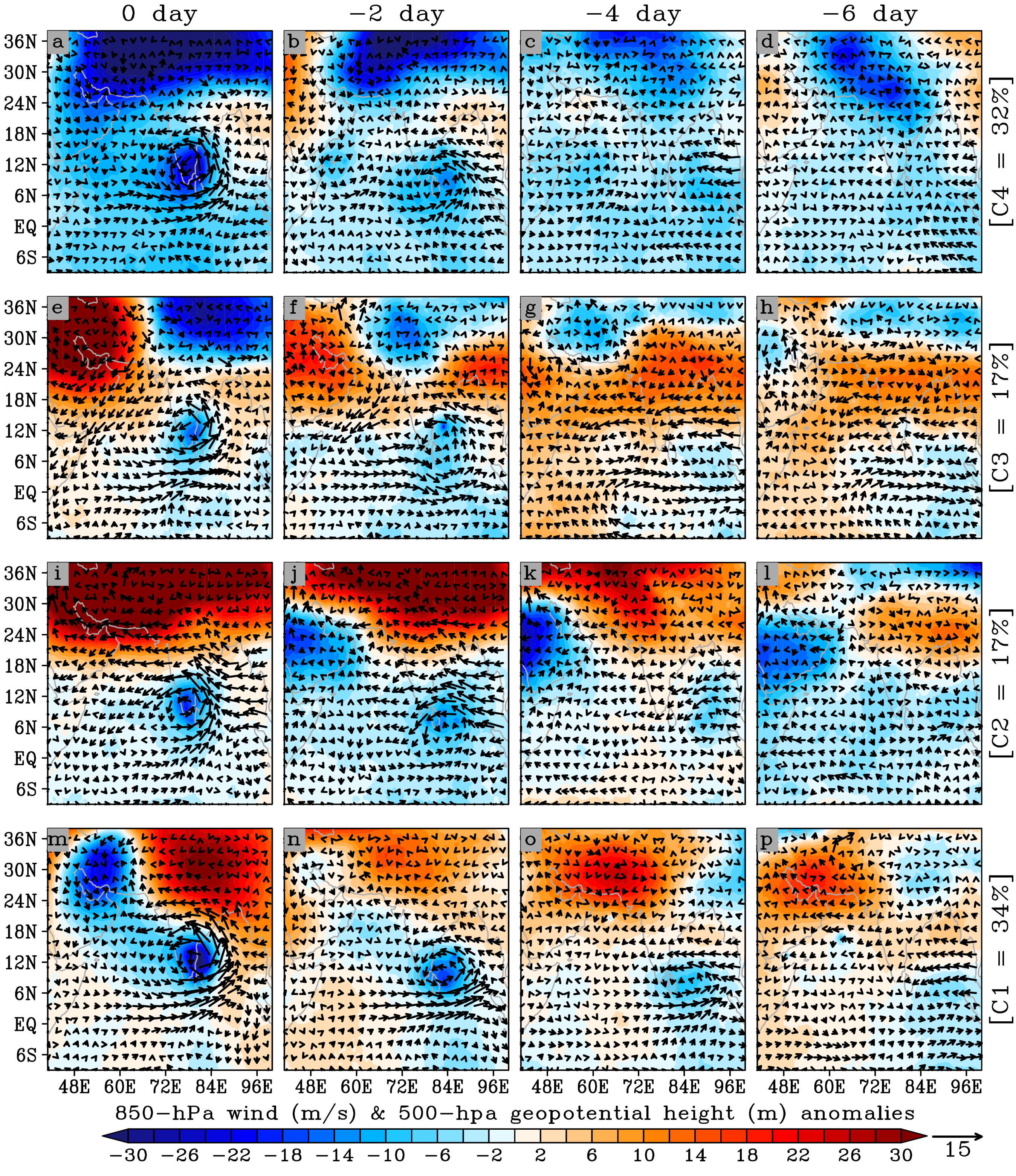

Fig. S1 K-means cluster patterns of 500-hPa geopotential height (in meters; shaded) along with 850-hPa wind vectors (in m/s; arrows) from IMDAA.

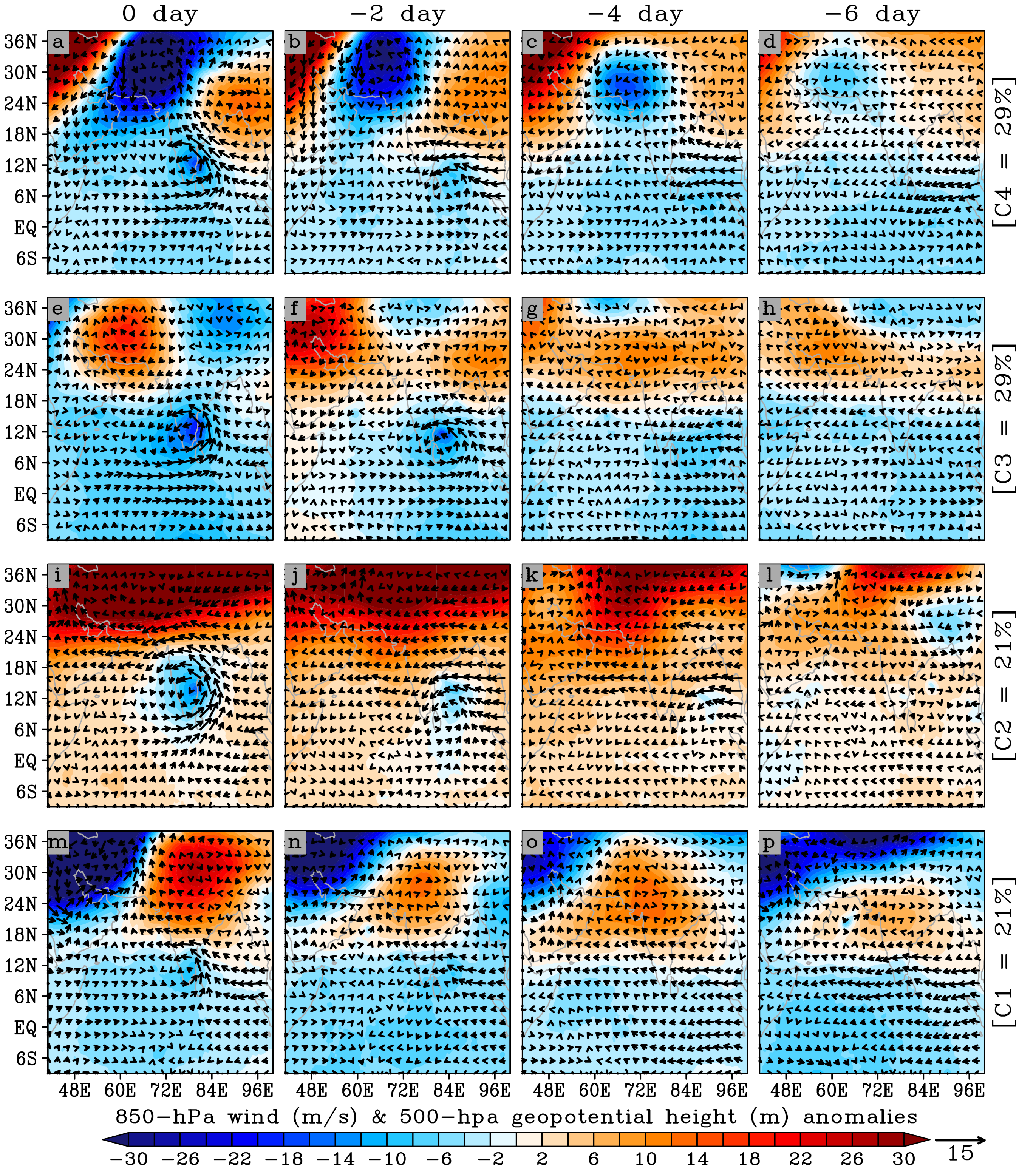

Fig. S2 K-means cluster patterns of 500-hPa geopotential height (in meters; shaded) along with 850-hPa wind vectors (in m/s; arrows) from CORDEX_MMM.
